# Alum as a Remedy in Croup

**Published:** 1871-01

**Authors:** 


Alum as a Remery in Croup.—This is an old but a very efficient one. It is usually given, about ten grains, once in ten minutes, until vomiting is induced, using at the same time, syrup of ipecac or the hive syrup freely—the latter subduing the inflammation, while alum has a more repulsive action.

